# Interaction of *MTHFR* polymorphism with *PAX1* methylation in cervical cancer

**DOI:** 10.1515/biol-2022-1052

**Published:** 2025-04-25

**Authors:** Xiao-Yan Zhou, Meng-Meng Chen, Jun-mei Yu, Yang Zhou, Yan-Song Luan, Bing-Qiang Zhang, Yun-Yuan Zhang

**Affiliations:** Department of Clinical Laboratory, The Affiliated Hospital of Qingdao University, Qingdao, Shandong, 266101, P.R. China; Qingdao Ruiside Medical Laboratory Co., LTD, 368 Hedong Road, High-tech Zone, Qingdao, Shandong, 266111, P.R. China

**Keywords:** cervical cancer, HR-HPV, *MTHFR*, *PAX1*, interaction effect

## Abstract

We aimed to investigate the roles and interaction effects of high-risk human papillomavirus (HR-HPV) infection, methyltetrahydrofolate reductase *(MTHFR)* polymorphism, and paired box gene 1 (*PAX1*) methylation in cervical intraepithelial neoplasia (CIN) and cervical cancer. Polymerase chain reaction was used to detect *MTHFR* polymorphism and *PAX1* methylation; Mantel–Haenszel and Spearman’s rank correlation tests were used to analyze the trends and correlations. Forty cases each of normal control (NC), CIN I, and CIN II/III and 9 squamous cell carcinoma (SCC) cases were enrolled. Increase in age increases the risk of cervical cancer. The HR-HPV infection rate, *MTHFR* mutation rate, and *PAX1* methylation rate in CIN I, CIN II/III, and SCC groups were significantly higher than those in the NC group (*P* < 0.05). The above-mentioned rates gradually increased with the degree of cervical lesions. Moreover, HR-HPV infection, *MTHFR* polymorphism, and *PAX1* methylation increased the risk of both CIN and cancer. A positive additive interaction was observed between *PAX1* methylation and *MTHFR* polymorphism across different cervical lesion groups, whereas no interaction was found between HR-HPV infection and *PAX1* methylation in lesion progression.

## 1 Introduction

Cervical cancer is a common gynecological malignancy and the fourth leading cause of cancer-related death among women worldwide [1]. In 2020, 604,127 new cases of cervical cancer and 341,831 deaths were estimated worldwide [2]. Thus, cervical cancer remains a global threat to women’s health. Most cervical cancer cases are usually curable if diagnosed early, with a 5-year overall survival rate of approximately 92% [3]. However, approximately 50% of the cases are diagnosed at a locally advanced stage, with a significant decrease in 5-year survival rate [3]. Therefore, early screening and treatment are of great importance for cervical cancer management.

Persistent infection by high-risk human papillomavirus (HR-HPV) is highly correlated with cervical cancer and cervical intraepithelial neoplasia (CIN) [4]; 99% of the patients with cervical cancer are estimated to be related to HR-HPV infection [5]. CIN, a precancerous lesion of cervical cancer, is also caused by HPV infection in cervical cells [6]. Low-grade cervical lesions are usually reversible [6]. Approximately 60% of CIN I are reported to regress to normal after a year due to an intact immune response and rapid turnover of cells in the cervix, especially in young women [6]. However, high-grade cervical lesions, such as CIN II/III, have a high risk of developing invasive cancer, although the average time of progression is still several years [6]. DNA methylation, the focus of epigenetics, is closely related to the occurrence and development of cancer [7,8,9]. High methylation in the promoter region of tumor suppressor genes is an early feature of various cancers [10]. The tumor suppressor paired box gene 1 (*PAX1*) was reported to present abnormal methylation in cervical cancer, and its methylation increased with disease grade, indicating the potential of *PAX1* methylation as a biomarker for cervical cancer screening [11]. Methyltetrahydrofolate reductase (*MTHFR*) is the rate-limiting enzyme in folic acid metabolism [12]. Variation in *MTHFR* can lead to insufficient methyl donors, thereby affecting cell functions associated with cancer risk [13]. For instance, compared to that in the control group, the frequency of rs4846048 AG and G alleles was significantly higher in the cervical cancer group [13]. A meta-analysis showed that *MTHFR* gene polymorphism (A1298C) is associated with increased susceptibility to cervical cancer in Asian populations, particularly under a recessive model [14]. Based on the above evidence, HR-HPV infection, *MTHFR* polymorphism, and DNA methylation of *PAX1* can all be considered risk factors for cervical cancer, although whether the three factors exert interaction effects in the process of cervical cancer remains elusive.

The current study represents the first exploration of the roles and interaction effects of HR-HPV infection, *MTHFR* polymorphism, and *PAX1* methylation in CIN. We hope that our findings will offer potential therapeutic avenues for preventing and treating CIN and cervical cancer.

## 2 Materials and methods

### 2.1 Patients

Nine patients with cervical squamous cell carcinoma (SCC) aged 34–63 years, 40 patients with CIN I aged 22–50 years, and 40 patients with CIN II/III aged 22–67 years, who were admitted to the Affiliated Hospital of Qingdao University between March and July 2023, were included in this study as the experimental groups. Forty normal controls (NCs) aged 21–67 years from the same period were designated as the control group. Criteria for inclusion of cases are as follows: (1) all cases diagnosed, grouped by cervical pathology, and verified by two senior pathologists; (2) healthy participants referred to as individuals who had undergone health screenings in the Huangdao Hospital of Affiliated Hospital of Qingdao University or those who have normal cervical pathology tests; (3) no case who received relevant drug treatment or surgery before inclusion in the study; and (4) all patients over 18 years of age. All participants signed informed consent. The NC and CIN I groups were matched proportionally to the CIN II/III group, with 40 participants each.


**Informed consent:** Informed consent was obtained from all individuals included in this study.
**Ethical approval:** The research related to human use complied with all the relevant national regulations, institutional policies, and in accordance with the tenets of the Helsinki Declaration, and the study was approved by the Ethics Committee of Affiliated Hospital of Qingdao University.

### 2.2 Screening and genotyping of HPV

Cervical exfoliated cells were collected and preserved in the cell preservation solution at 4°C. All participants were abstained from sexual intercourse, vaginal medication, and douching 72 h prior to collection. The screening and genotyping of HPV were completed using quantitative fluorescence polymerase chain reaction (PCR), which could identify 13 types of HR-HPV.

### 2.3 Extraction of DNA samples

Total DNA samples from cervical exfoliated cells were extracted using the Swab Genomic DNA Kit (CoWin Biosciences, Jiangsu, China) following the supplier’s protocols. The extracted DNA sample concentration was determined using the Nano-500 spectrophotometer (Allsheng, Hangzhou, China). The DNA samples were stored at −20°C.

### 2.4 Bisulfite modification of DNA samples

The extracted DNA samples were subjected to bisulfite modification using the EpiArt^®^ DNA Methylation Bisulfite Kit (Vazyme, Nanjing, China) following the supplier’s protocols. The bisulfite-modified DNA was immediately used for dual fluorescence quantitative PCR, and the remaining DNA samples were stored at −20°C.

### 2.5 Detection of MTHFR C677T polymorphism using fluorescence quantitative PCR

The detection of single nucleotide polymorphism (SNP) of *MTHFR* C677T in 129 subjects was performed using fluorescence quantitative PCR. It was conducted in a 20 μl reaction system on the ABI 7300 System. The reaction system included 10 μl of 2× ChamQ Geno-SNP Probe Master Mix, 1.8 μl of forward primer (10 μM), 1.8 μl of reverse primer (10 μM), 2 μl of DNA template, 0.4 μl of MGB probe, and 4 μl of deionized water. The primers of *MTHFR* C677T sites were as follows:

F, 5′-CAAAGAAAAGCTGCGTGATGAT-3′;

R, 5′-GACCTGAAGCACTTGAAGGAGAA-3′.

### 2.6 Dual fluorescence quantitative PCR

Methylation of *PAX1* in cervical exfoliated cells was detected by dual fluorescence quantitative PCR on the ABI 7500 System. Hypermethylated and hypomethylated *PAX1* standard samples and sterile water were used as the positive, negative, and blank control templates, respectively. PCR was conducted in a 20 μl reaction system, which included 2.8 μl of 10× TaqHs mix, 0.4 μl of forward primer of *PAX1*, 0.4 μl of reverse primer of *PAX1*, 0.4 μl of forward primer of *ACTB*, 0.4 μl of reverse primer of *ACTB*, 0.2 μl of *PAX1* probe, 0.2 μl of *ACTB* probe, 2 μl of DNA template, and 13.2 μl of deionized water. The PCR program was as follows: 30 s at 95°C for pre-denaturation, 45 cycles of 10 s at 95°C for denaturation, and 30 s at 60°C for annealing and extension. After collecting fluorescence signals on the ABI 7500, the cycle threshold (CT) values and amplification curves of the internal reference gene and target gene were obtained, the difference in CT values between *PAX1* and *ACTB* was calculated (ΔCt value), and the degree of methylation in different samples was determined. *ACTB* was designated as an internal reference gene. The primer and probe sequences of *PAX1* and *ACTB* are shown in Table 1.

**Table 1:** List of primers used for quantitative PCR

Genes	Sequences (5′−3′)	Length (bp)
PAX1-F	TTTTGGTATTTTTGTTTGGGAGATT	25
PAX1-R	TCTCCCAAACAAAAATACCAAAATCTC	27
PAX1-P	ACCAATATAAAACCCTCCCCTAAACC	26
ACTB-F	TGTGTGATTCGGAGTGCATG	20
ACTB-R	TCAGATGAACAAGCCTAACTC	21
ACTB-P	CCACCCTATCATCATCATATTAAC	24

### 2.7 Statistical analysis

Data analysis was completed using SPSS Statistics 26.0 (SPSS, Chicago, IL, USA). Categorical variables were expressed as counts (percentages), and differences between groups were compared using the chi-squared test. First, the Shapiro–Wilk test was used to check the distribution of continuous variables. The results are presented as mean ± standard deviation for data following a normal distribution. Comparisons between two groups were performed using the independent Student’s *t*-test, while comparisons across three or more groups were conducted using the *F*-test. For data following a non-normal distribution, the results are presented as median (interquartile range), and differences between groups were analyzed using the non-parametric Mann–Whitney *U* test. The Mantel–Haenszel test was used to analyze linear trends. The Spearman’s rank correlation test was used to assess the correlation between *MTHFR* polymorphism and *PAX1* methylation. The interactions between *MTHFR* polymorphism and *PAX1* methylation rate were assessed using additive measures to investigate their association with cervical lesions. Additive interaction was evaluated using three metrics, namely the relative excess risk due to interaction (RERI), calculated as RERI = OR_AB_ – (OR_A_ + OR_B_) + 1; the attributable proportion due to interaction (AP), calculated as AP = RERI/OR_AB_; and the synergy index (*S*), calculated as *S* = (OR_AB_ – 1)/[(OR_A_ – 1) + (OR_B_ – 1)]. If the RERI was greater than 0, so was AP, and the *S* was greater than 1, thus indicating the presence of a positive additive interaction. Conversely, if RERI equaled 0, AP equaled 0, or *S* equaled 1, the absence of an additive interaction would be indicated. *P* < 0.05 was identified as the criterion for statistical significance.

## 3 Results

### 3.1 Risk of cervical cancer increased with age

A total of 129 subjects were enrolled in this study, including 40 NC, 40 CIN I, 40 CIN II/III, and 9 SCC cases. Given that HR-HPV subtype HPV16 infection is the primary type leading to cervical cancer, this study defined HR-HPV16 infection as HR-HPV positive. In the NC, CIN I, CIN II/III, and SCC groups, 16 (40%), 32 (80%), 35 (87.5%), and 8 (88.9%) subjects, respectively, tested positive for HR-HPV infection. Age differences among the groups were significant, and increasing age increased the risk of cervical cancer. Ages of subjects in the NC, CIN I, CIN II/III, and SCC groups were 39.33 ± 10.33 years, 35.23 ± 10.07 years, 43.18 ± 10.99 years, and 53.11 ± 12.96 years, respectively, and the differences in ages across the four groups were significant (*P* < 0.001, Table 2). The results indicated that an increase in age increased the risk of cervical cancer.

**Table 2:** Age distribution of subjects among different cervical lesion groups

Factor	NC	CIN Ⅰ	CIN II/III	SCC	*χ* ^2^ value	*P* value
Age, *n*					27.544	0.001
<35	14	22	11	2		
35∼	10	9	11	0		
45∼	14	7	10	2		
>55	2	2	8	5		
Sum	40	40	40	9		
Mean age, year	39.33 ± 10.33	35.23 ± 10.07	43.18 ± 10.99	53.11 ± 12.96		<0.0001
HR-HPV infection	16 (40%)	32 (80%)	35 (87.5%)	8 (88.9%)		<0.0001

### 3.2 Infection rate of HR-HPV gradually increased with the severity of cervical lesions

**Figure 1:**
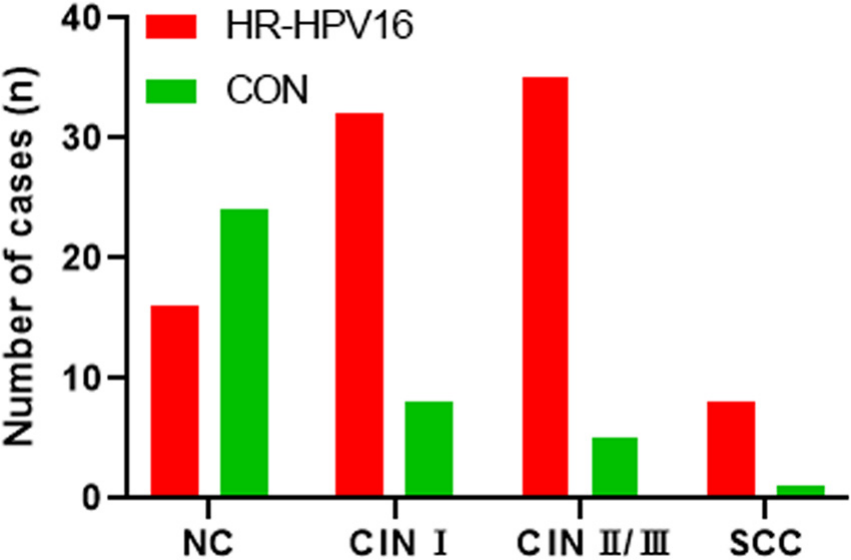
HR-HPV infection in different cervical lesion groups. The HR-HPV16 infection cases in CIN I, CIN II/III, SCC, and NC groups were determined. The green bars represent the control group, which consists of patients without CIN. The red bars indicate HR-HPV16 (high-risk human papillomavirus type 16) positive cases.

The HR-HPV infection rate was found to be significantly higher in the CIN I (32 cases, 80%) (*P* < 0.01), CIN II/III (35 cases, 88%) (*P* < 0.05), and SCC groups (8 cases, 89%) (*P* < 0.05) than in the NC group (16 cases, 40%) (Figure 1). As cervical lesions progressed, the infection rate and odds ratio (OR) value of HR-HPV gradually increased (trend test: *χ* ^2^ = 20.928, *P* < 0.05) (Table 3). Overall, the HR-HPV infection rate gradually increased with the severity of cervical lesions.

**Table 3:** Analysis of the relationship between HR-HPV infection and cervical lesions

Factor	Case	HR-HPV infection *n* (%)	OR (95% CI)	aOR (95% CI)
NC	40	16 (40%)	1	1
CIN I	40	32 (80%)	6 (2.207–16.313)	2.683 (0.7–10.281)
CIN Ⅱ/III	40	35 (88%)	10.5 (3.39–32.523)	6.708 (1.02–37.444)
SCC	9	8 (89%)	12 (1.366–105.412)	4.083 (0.412–40.455)
*χ* ^2^ = 26.671, *P* < 0.05; trend test: *χ* ^2^ = 20.928, *P* < 0.05				

Note: “a” refers to the adjusted OR and 95% CI for MTHFR in CIN I, CIN II/III, and cervical cancer group, respectively.

### 3.3 MTHFR mutation rate increased with the severity of cervical lesions

**Figure 2:**
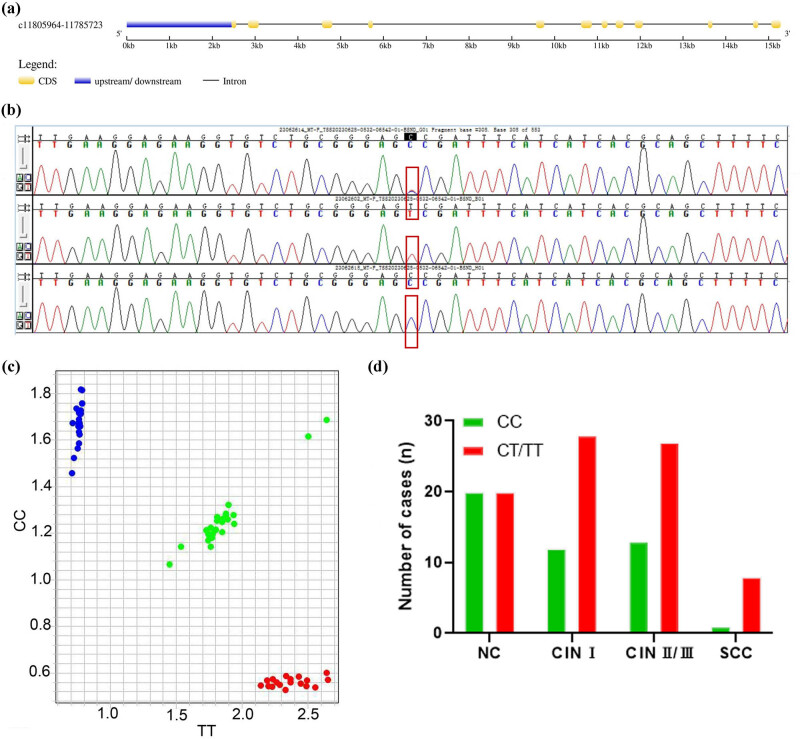
Analysis of the relationship between *MTHFR* (C677T) polymorphism and cervical lesions. (a) Gene structure of *MTHFR*. (b) *MTHFR* gene sequence alignment diagram. Blue peaks represent the CC genotype and red peaks represent the TT genotype. Red boxes indicate the mutation site. (c) *MTHFR* gene identification map. Blue dots represent the CC genotype, red dots represent the TT genotype, and green dots represent the CT genotype. (d) Number of *MTHFR* mutation cases in CIN I, CIN II/III, SCC, and NC groups.

*MTHFR* is a critical gene in folate acid metabolism; it is located on human chromosome 1p36.3, with a total length of 20.2 kb (chr1:11805964–11785723) and consisting of 12 exons (Figure 2a). If *MTHFR* (C677T) polymorphism leads to alanine valine substitution, it can reduce the activity of the MTHFR enzyme. In this study, in the *MTHFR* genotype, CC is the wild-type [15] and the CT or TT genotype is the mutant [16] (Figure 2b and c). *MTHFR* mutation rates were determined in different cervical lesion groups. The results showed that 28 in the CIN I group were mutant type (70%), 27 in the CIN II/III group were mutant type (68%), 8 in the SCC group were mutant type (89%), and 20 in the NC group were mutant type (50%) (Figure 2d). The wild-type and mutant *MTHFR* gene distribution in cervical lesion samples exhibited statistical differences (*χ* ^2^ = 7.932, *P* < 0.05, Table 4). Overall, the results indicated that *MTHFR* mutation rate increased with the severity of cervical lesions.

**Table 4:** Analysis of the relationship between MTHFR (C677T) polymorphism and cervical lesions

Factor	*MTHFR*		Sum	*χ* ^2^ value	*P* value
	Wild (CC)	Mutant (CT/TT)			
Cervical lesions				4.359	0.037
NC	21	19	40		
CIN Ⅰ	12	28	40		
CIN Ⅱ/III	14	26	40		
SCC	1	8	9		
Sum	48	81	129		

### 3.4 DNA methylation rate of PAX1 increased with the severity of cervical lesions

DNA methylation of *PAX1* was investigated, but no *PAX1* methylation was detected in the NC group, and 20 cases each of *PAX1* methylation were detected in CIN I (50%) and CIN II/III samples (50%). *PAX1* methylation was found in all 9 cases of SCC. Therefore, the *PAX1* methylation status significantly correlated with the severity of cervical lesions (*χ* ^2^ = 44.097, *P* < 0. 001) (Table 5).

**Table 5:** Analysis of the relationship of the DNA methylation rate of *PAX1* and cervical lesions

Factor	PAX methylation		Total	*χ* ^2^ value	*P* value
	Yes	No			
Cervical lesions				44.097	*P* < 0.001
NC	0	40	40		
CIN I	20	20	40		
CIN II/III	20	20	40		
SCC	9	0	9		
Total	49	80	129		
Trend test: *χ* ^2^ = 36.044, *P* < 0.001					

### 3.5 Interaction among HR-HPV infection, MTHFR polymorphism, and PAX1 methylation

HR-HPV infection, *MTHFR* polymorphism, and DNA methylation of *PAX1* are all risk factors for cervical cancer. However, whether the three factors have interaction effects on the progression of cervical cancer remains elusive. Therefore, the interaction effect among HR-HPV infection, *MTHFR* polymorphism, and methylation of *PAX1* was determined using the *F*-test. The results showed no interaction effect between HR-HPV infection and *PAX1* methylation on the progression of cervical lesions (F (1,118) = 0.260, *P* = 0.611, Table 6). However, *MTHFR* polymorphism and *PAX1* methylation did have an interaction effect on the progression of cervical lesions (F (2,118) = 6.299, *P* < 0.05, Table 6).

**Table 6:** Interaction between HR-HPV infection, *MTHFR* polymorphism, and *PAX1* methylation

Factor	df	*F* value	*P* value
HR-HPV infection * *PAX1* methylation	1	0.260	0.611
*MTHFR* polymorphism * *PAX1* methylation	2	6.299	0.001

### 3.6 Additive interaction effect between MTHFR polymorphism and PAX1 methylation on the progression of cervical lesions

**Figure 3:**
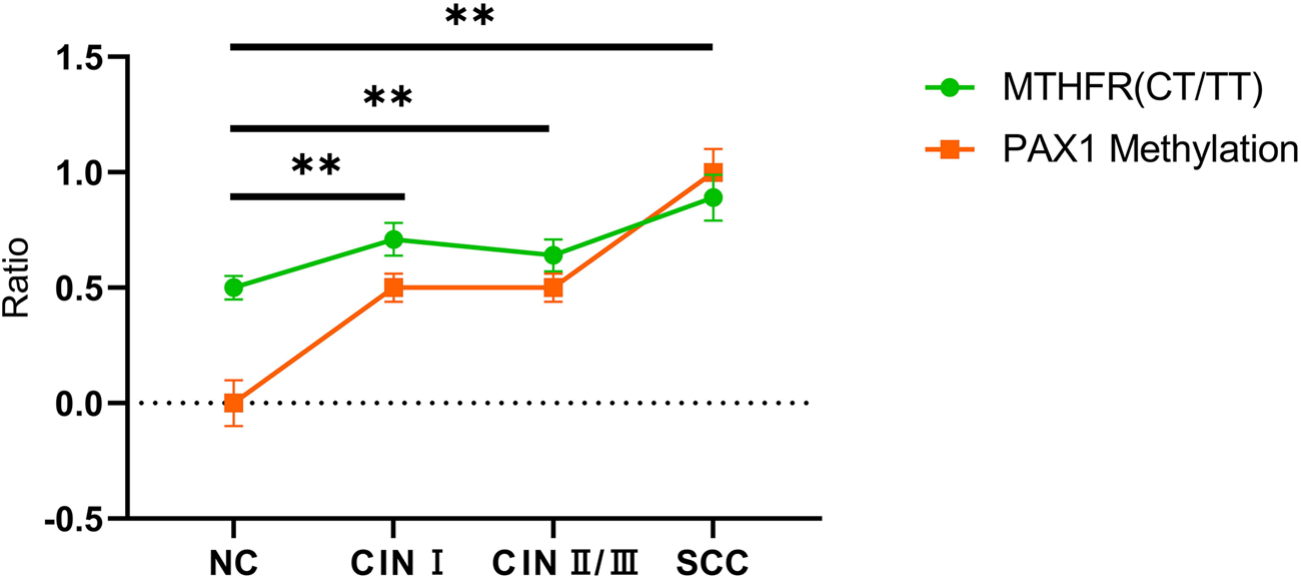
Interaction between *MTHFR* polymorphism and *PAX1* methylation. The correlation between *MTHFR* polymorphism and *PAX1* methylation was assessed using Spearman’s rank correlation analysis. **P < 0.01.

To further clarify the effect of interaction between *MTHFR* polymorphism and *PAX1* methylation rate on the progression of cervical lesions, the Spearman rank correlation test was performed; the results revealed that *MTHFR* polymorphism positively correlated with the methylation of *PAX1* (*r* = 0.458, *P* < 0.001, Figure 3). Additionally, the additive model was applied to determine the interaction effect between *MTHFR* polymorphism and *PAX1* methylation on the progression of cervical lesions. No interaction between *MTHFR* polymorphism and *PAX1* methylation was found in the CIN I group, and the interaction indicators EREI, AP, and S were 0, 0, and 1, respectively (Table 7). Interestingly, in both CIN II/III and SCC groups, the additive interaction effect between *MTHFR* polymorphism and *PAX1* methylation was positively significant with RERI (CIN II/III group: 0.8, SCC group: 0.5), AP (CIN II/III group: 0.8, SCC group: 1.2), and S (CIN II/III group: 0, SCC group: 0.5) (Table 7). Hence, the additive interaction effect between *MTHFR* polymorphism and *PAX1* methylation in cervical lesions was confirmed.

**Table 7:** Additive interaction between *MTHFR* polymorphism and *PAX1* methylation

Factor	*MTHFR* polymorphism	*PAX1* methylation	OR (95% CI)	aOR (95% CI)	RERI	AP	*S*	*P* value
CIN Ⅰ	−	−	1	1	0	0	1	0.327
	−	+	0.647 (0.166–2.527)	0.657 (0.278–1.552)				
	+	−	0.824 (0.434–1.561)	1.131 (0.891–1.436)				
	+	+	1.273 (0.614–2.64)	1.721 (0.578–5.124)				
CIN Ⅱ/III	−	−	1	1	0.8	0.8	0	0.019
	−	+	1.797 (0.575–2.105)	1.538 (0.308–1.937)				
	+	−	3.333 (0.692–16.055)	1.608 (1.076–2.405)				
	+	+	4.182 (0.662–26.415)	2.99 (1.184–7.554)				
SCC	−	−	1	1	0.5	1.2	0.5	0.033
	−	+	1.143 (0.88–1.485)	1.136 (0.019–0.995)				
	−	−	1.533 (1.12–1.998)	1.432 (1.08–1.898)				
	+	+	10.7 (1.312–90.021)	10.5 (1.211–91.026)				

Note: MTHFR+: CC; MTHFR−: CT/TT; PAX1+: methylated; PAX1−: not methylated; “a” refers to the adjusted OR and 95% CI for MTHFR in CIN I, CIN II/III, and cervical cancer groups, respectively.

## 4 Discussion

Cervical cancer ranks among the most commonly diagnosed cancers and is a leading cause of cancer-related mortality in women [17]. Unfortunately, the majority of cases are identified at the advanced stage, resulting in poor prognosis [18]. In China, the age-standardized incidence rate and mortality of cervical cancer are rising rapidly [19]. Despite recent advancements in surgical techniques, radiotherapy, and chemotherapy, improvements in the 5-year survival rate have been limited, and long-term survival rates remain unsatisfactory [20]. Therefore, investigating the mechanism of cervical cancer and searching for early screening and treatment therapy for patients have become essential. The progression of cervical cancer typically follows a sequence from normal epithelium to low-grade squamous intraepithelial lesions, then to high-grade lesions, carcinoma *in situ*, and eventually metastatic cancer [21]. High-risk HPV infection, along with genetic and epigenetic changes, is closely linked to the pathogenesis and malignant transformation of cervical cancer [22]. During this complex tumorigenesis, DNA methylation occurs early and is the most common molecular event, with alterations accumulating with the progression of the disease, serving as precursors to malignancy [23,24]. Epidemiological studies have identified *MTHFR* as a potential genetic marker for various cancers [25]. *MTHFR* encodes a crucial enzyme in the folate/homocysteine metabolic pathway, regulating intracellular folate levels necessary for DNA synthesis and methylation [26]. However, the interaction effects of these factors on cervical cancer development remain unclear. In this study, we explored the roles and interactions of HR-HPV infection, *MTHFR* polymorphism, and *PAX1* methylation in CIN and cervical cancer. This is the first study to investigate the interaction effects of *PAX1* methylation and *MTHFR* polymorphism on cervical lesion progression. The findings indicate that HR-HPV infection, *MTHFR* polymorphism, and *PAX1* methylation increase the risk of cervical cancer and its precursors, with *MTHFR* polymorphism and *PAX1* methylation showing an additive interaction effect on cervical lesion development.

The pathogenesis of cervical cancer is complex, and persistent infection by HR-HPV is closely related to the onset of cervical cancer and CIN [27]. Therefore, we explored the relationship between HR-HPV infection and cervical lesions in different groups. The results revealed that the HR-HPV infection rate gradually increased with the severity of cervical lesions, and the HR-HPV infection rate in the cervical cancer group was the highest at 89%. This finding was consistent with that reported previously [28]. Li et al. reported that the infection rate of HR-HPV increased with the severity of cervical histological lesions [28].

Epigenetic research indicates that DNA methylation is a key event in carcinogenesis [29]. Various types of DNA methylation, such as those involving *PAX1*, *ZNF582*, and *FAM19A4*, are strongly linked to CINs and cervical cancer [11,30,31]. In cervical cancer, the *PAX1* gene, which acts as a tumor suppressor, is silenced through methylation [11]. Numerous studies have confirmed that *PAX1* methylation is highly correlated with the progression of CIN and cervical carcinogenesis [32–34]. Epidemiological research has identified *MTHFR* as a potential genetic marker for various cancers [12]. Located on chromosome 1p36.3, *MTHFR* is a crucial enzyme in folic acid metabolism, playing a significant role in the folate/homocysteine metabolic pathway and regulating intracellular folate levels for DNA synthesis and methylation [35]. Increasing evidence suggests that *MTHFR* polymorphisms are associated with cervical cancer susceptibility [13,25,36]. Zhou et al. found that the rs4846048 AG genotype and G allele frequencies were significantly higher in the cervical cancer subgroup than in the controls [13]. In this study, we examined *PAX1* methylation and *MTHFR* polymorphisms in cervical exfoliated cells. Our results indicated that both *MTHFR* mutation and *PAX1* methylation rates increased with the severity of cervical lesions. The findings collectively suggested that *PAX1* methylation and *MTHFR* polymorphism might have an interaction effect on the occurrence and progression of cervical cancer.

The present study provided compelling evidence of the interaction between *MTHFR* polymorphisms and *PAX1* methylation on the progression of cervical lesions, specifically CIN II/III and cervical cancer. Statistical analysis revealed a significant interaction effect, with *F* (2,118) = 6.299 and a *P*-value < 0.05, highlighting the importance of considering these factors jointly rather than in isolation. The presence of additive interaction, particularly in the context of CIN II/III and cervical cancer progression, underscores the heightened risk conferred when both *MTHFR* polymorphisms and *PAX1* methylation are present. This additive effect was quantitatively supported using interaction effect indicators, such as RERI, AP, and SI, which progressively increased with the severity of cervical lesions. This trend suggested that the synergistic interaction between *PAX1* methylation and *MTHFR* polymorphism becomes more pronounced with the advancement of cervical lesions, potentially exacerbating the risk of progression to malignancy. The methylation status of the *PAX1* gene is associated with the development of various cancers, including cervical cancer [33]. Methylation is typically linked to gene silencing, but in certain cases, such as with *PAX1*, it may lead to gene overexpression. *MTHFR* is a key enzyme involved in folate metabolism and DNA methylation processes [37]. Mutations in *MTHFR*, such as C677T, can disrupt folate metabolism, affecting the availability of DNA methyl donors [38]. Thus, *MTHFR* mutations are expected to reduce methyl donors, leading to *PAX1* hypomethylation. Contrary to expectations, our data indicate that high methylation of the *PAX1* gene is associated with *MTHFR* mutations, which may be due to several factors: 1. alternative methylation pathways or compensatory mechanisms might be upregulated when *MTHFR* function is impaired, maintaining or increasing methyl donor availability. 2. Epigenetic regulation is complex, involving multiple molecules and signaling pathways. There may be unidentified molecules or pathways regulating *PAX1* methylation in the context of *MTHFR* mutations. 3. Our sample may possess specific genetic or environmental backgrounds influencing the relationship between *MTHFR* mutations and *PAX1* methylation. Therefore, further research is needed to validate our results in larger populations. Additional molecular biology experiments, including gene expression analysis and protein–DNA interaction studies, are necessary to explore the mechanisms underlying the interaction between *PAX1* methylation and *MTHFR* polymorphism. Environmental factors such as diet and lifestyle should also be considered to better understand the observed interaction. Furthermore, we plan to conduct long-term follow-up studies to assess the impact of *PAX1* methylation and *MTHFR* polymorphisms on disease progression and treatment response over time.

This study has several limitations. First, the clinical sample size was relatively small, particularly regarding the number of patients with SCC, which might limit the statistical power and generalizability of the findings. Second, the participants, comprising NC and patients with CIN I, CIN II/III, or SCC, were all from a single hospital and limited to a Chinese population. Therefore, the findings may not be generalizable to a broader population. Consequently, further large-scale prospective studies would be required to validate the results of this study.

In conclusion, the results indicate that HR-HPV infection, *MTHFR* mutations, and *PAX1* methylation increase the risk of CIN and cervical cancer. Additionally, *MTHFR* mutations and *PAX1* methylation have an interaction effect on cervical lesion development. However, HR-HPV and *PAX1* methylation did not interact in the progression of cervical lesions. These findings provided new insights for the early detection, prevention, and treatment of cervical lesions. However, further large-scale prospective studies would be required to validate the results.
